# Supplementary material for: Brain clusterin protein isoforms and mitochondrial localization
Source: eLife. 2019 Nov 18;8:e48255. doi: 10.7554/eLife.48255 (PMC6860991; doi:10.7554/eLife.48255)
Supplement: Supplementary file 1. [file elife-48255-supp1.pdf]

IDEXX BioAnalytics

FINAL REPORT OF LABORATORY EXAMINATION

4011 Discovery Drive, Columbia, MO 65201
1-800-669-0825 1-573-499-5700
idexxbioanalytics@idexx.com www.idexxbioanalytics.com

IDEXX BioAnalytics Case # 24482-2019 **Received: 8/27/2019**
Client Case # 20190826 **Completed: 8/28/2019**

Submitted By

Liqin Zhao
University of Kansas
1251 Wescoe Hall Dr.
Lawrence , KS 66045
Phone: 785-864-4088
Email: lzhao@ku.edu

Specimen Description

Species: human/mouse
Description: Cells
Number of Specimens/Animals: 2

ID	Client ID	Species
1	N2a	mouse
2	SH-SY5Y	human

Services/Tests Performed: CellCheck 9 - human (9 Marker STR Profile and Inter-species Contamination Test) (2); CellCheck - mouse (mouse STR profile and interspecies contamination test) (1)

Genetic evaluation for: Human 9-Marker STR Profile, Interspecies Contamination Test, Interspecies Contamination Test, Mouse STR Profile

Summary: Cell Check results are provided in the data results section for each sample. For human samples, an identity matching score above 80% indicates the sample is consistent with the cell line of origin. For human samples with less than an 80% matching score, please see individual comments for these samples in the detail section. For all other species, please see individual detailed results.

Please see the report for details.

CELL CHECK

Species-specific PCR Evaluation

Species	1
mouse	+
rat	-
human	-
Chinese hamster	-
African green monkey	-

Marker Analysis

Marker Name	1	
	Sample Results	Neuro 2A (IBA Profile)
MCA-4-2	21.3, 22.3	21.3, 22.3
MCA-5-5	15, 17	15, 17
MCA-6-4	18, 20	18, 20
MCA-6-7	12	12
MCA-9-2	15, 16	15, 16
MCA-12-1	16	16
MCA-15-3	21.3, 22.3, 23.3	21.3, 22.3, 23.3
MCA-18-3	22	22
MCA-X-1	26, 27	26, 27

Sample ID	Remarks
1	The sample was confirmed to be of mouse origin and no mammalian interspecies contamination was detected. A genetic profile was generated for the sample by using a panel of STR markers for genotyping.<br><br>The sample profile matches identically to the genetic profile established for this cell line.

Species-specific PCR Evaluation

Species	2
mouse	-
rat	-
human	+
Chinese hamster	-
African green monkey	-

Marker Analysis

Marker Name	2	
	Sample Results	SH-SY5Y (ATCC# CRL-2266)
AMEL	X	X
CSF1PO	11	11
D13S317	11	11
D16S539	8, 13	8, 13
D5S818	12	12
D7S820	7, 10	7, 10
TH01	7, 10	7, 10
TPOX	8, 11	8, 11
vWA	14, 18	14, 18
Identity Match	100%	
