# Supplementary material for: Brain clusterin protein isoforms and mitochondrial localization
Source: eLife. 2019 Nov 18;8:e48255. doi: 10.7554/eLife.48255 (PMC6860991; doi:10.7554/eLife.48255)
Supplement: Supplementary file 2. [file elife-48255-supp2.pdf]

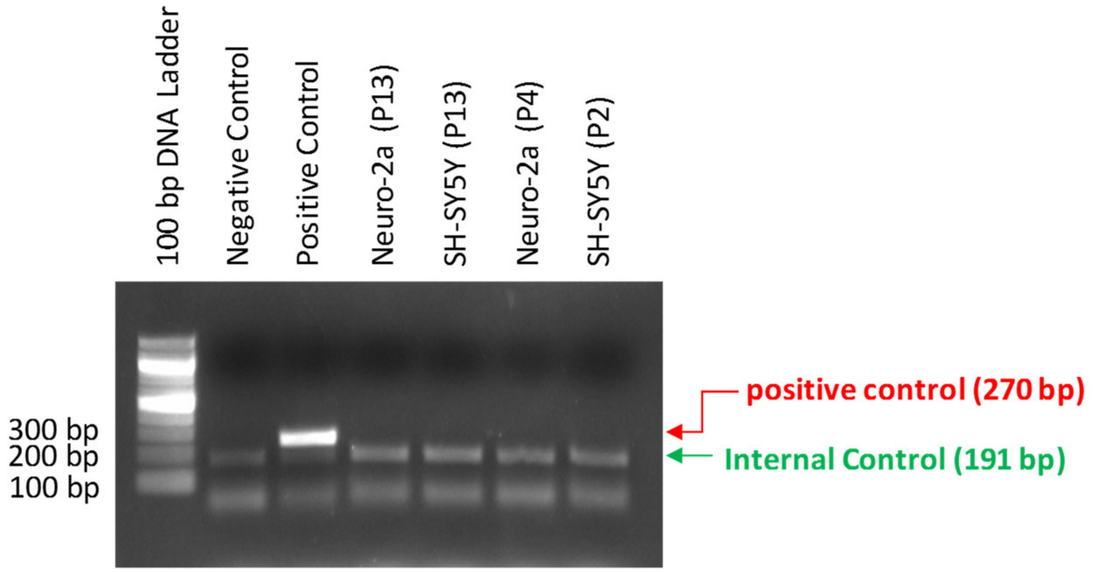


**Mycoplasma testing of cell lines at different passages.** Neuro-2a (P4 and P13) and SH-5YSY (P2 and P13) cells from different passages were tested for mycoplasma contamination. Negative results indicate no mycoplasma contamination was present in the cell lines used in the studies presented herein.
